# The lncRNAs RP1-261G23.7, RP11-69E11.4 and SATB2-AS1 are a novel clinical signature for predicting recurrent osteosarcoma

**DOI:** 10.1042/BSR20191251

**Published:** 2020-01-06

**Authors:** Tang Ying, Jin-ling Dong, Cen Yuan, Peng Li, Qingshan Guo

**Affiliations:** 1 Trauma Center, State Key Laboratory of Trauma, Burns and Combined Injury, Institute of Surgery Research, Daping Hospital, Army Medical University, No.10 ChangjiangZhi Road, Yuzhong District, Chong Qing 400042, China; 2 Department of Pharmacognosy and Traditional Chinese Medicine, Department of Pharmacy and Laboratory Medicine, Army Medical University, No.30 Gaotanyan Centre Street, Shapingba District, Chong Qing 400038, China

**Keywords:** biomarker, lncRNA, osteosarcoma, recurrence rates

## Abstract

**Background:** Osteosarcoma is the most common primary bone malignancy in children and adolescents. In order to find factors related to its recurrence, and thus improve recovery prospects, a powerful clinical signature is needed. Long noncoding RNAs (lncRNAs) are essential in osteosarcoma processes and development, and here we report significant lncRNAs to aid in earlier diagnosis of osteosarcoma.

**Methods:** A univariate Cox proportional hazards regression analysis and a multivariate Cox regression analysis were used to analyze osteosarcoma patients’ lncRNA expression data from the Therapeutically Applicable Research To Generate Effective Treatments (TARGET), a public database.

**Results:** A lncRNA signature consisting of three lncRNAs (RP1-261G23.7, RP11-69E11.4 and SATB2-AS1) was selected. The signature was used to sort patients into high-risk and low-risk groups with meaningful recurrence rates (median recurrence time 16.80 vs. >128.22 months, log-rank test, *P*<0.001) in the training group, and predictive ability was validated in a test dataset (median 16.32 vs. >143.80 months, log-rank test, *P*=0.006). A multivariate Cox regression analysis showed that the significant lncRNA was an independent prognostic factor for osteosarcoma patients. Functional analysis suggests that these lncRNAs were related to the PI3K-Akt signaling pathway, the Wnt signaling pathway, and the G-protein coupled receptor signaling pathway, all of which have various, important roles in osteosarcoma development. The significant 3-lncRNA set could be a novel prediction biomarker that could aid in treatment and also predict the likelihood of recurrence of osteosarcoma in patients.

## Introduction

Osteosarcoma is a common malignant bone tumor, which is predominant in childhood and adolescence [1]. Multidrug chemotherapy and surgical removal of primary tumors have shown ameliorating effects [2]. Although improvements in therapeutic strategies have been achieved, the prognosis remains poor for most patients with recurrent osteosarcoma. It is therefore imperative to identify effective and novel recurrent biomarkers and therapeutic targets for osteosarcoma.

Long noncoding RNAs (lncRNAs) are a novel class of RNA molecules, defined as transcripts >200 nucleotides that lack protein-coding potential. There have been many studies on the mechanistic role of lncRNA in osteosarcoma. Dysregulation of lncRNAs is implicated in different physiological processes in osteosarcoma, such as cell growth and metastasis [3–5]. For example, nine lncRNAs (91H, FGFR3-AS1, BCAR4, TUG1, UCA1, HIF2PUT, HOTTIP, HULC, and MALAT-1) are up-regulated in osteosarcoma, which is considered oncogenic for osteosarcoma [6]. The lncRNAs MALAT1 and HNF1A-AS1 promote cell proliferation and metastasis in osteosarcoma by activating the PI3K/Akt [7] and Wntβ-catenin [8] signaling pathways, respectively. Moreover, lncRNA TUG1 down-regulation inhibits proliferation of osteosarcoma cells and promotes apoptosis [9].

There have also been many lncRNA studies focusing on clinical prognosis in osteosarcoma. Dysregulation of lncRNA MEG3 could act as a potential predictor of the progression and poor prognosis of osteosarcoma [10], and overexpression of lncRNAs UCA1 and TUG1 are essential in the poor prognosis of osteosarcoma [11,12]. In addition, SNHG4 promotes tumor growth and is a predictor of poor survival and recurrence in human osteosarcoma [13]. HNF1A-AS1 is a negative prognostic biomarker that promotes tumorigenesis and progression in osteosarcoma [14]. However, prognostic signatures of recurrence are still rare, meaning that the identification of such signatures is necessary to improve clinical treatment.

In the present study, we identified an lncRNA signature in osteosarcoma from the Therapeutically Applicable Research To Generate Effective Treatments (TARGET) database, and the signature was an independent prognostic factor for osteosarcoma. The presence of the signature allowed patients to be separated into high-risk and low-risk groups, and this signature may enable the development of novel recurrence biomarkers and therapeutic targets for osteosarcoma.

## Materials and methods

### Clinical cohorts and RNA-Seq data

Clinical information (age, gender) of 92 patients with osteosarcoma was obtained from the TARGET data portal (https://ocg.cancer.gov/), including 47 recurrent samples, 32 non-recurrent samples, and 13 censored samples. The RNA expression data (level 3) of 92 osteosarcoma samples were downloaded from the freely available TARGET data portal. The RNA sequence data from the 92 samples was derived from the IlluminaHiSeq_RNASeq sequencing platforms, and the differentially expressed mRNAs and lncRNAs were obtained by using the edgeR software [15] to further analyze the recurrence vs. non-recurrence data. Fold changes (log_2_ absolute) ≥2 and *P*<0.05 indicated a statistically significant difference.

### Construction of co-expression network

We examined the correlation between the expression levels of the 42 differentially expressed lncRNAs and each differentially expressed mRNA using two-sided Pearson correlation coefficients and the z-test. The mRNAs positively or negatively correlated with the 42 lncRNAs were considered to be lncRNA-related mRNAs (|Pearson correlation coefficient| > 0.4 [16–20] and *P*-value <0.05). The lncRNA-mRNA co-expression network was then constructed using Cytoscape V.3.8.5 (https://cytoscape.org/).

### Construction of an lncRNA signature in the training dataset

We performed a univariate Cox proportional hazards regression analysis [21] to assess the relationship between patient recurrence and differentially expressed lncRNAs in the training group. In order to screen the most powerful diagnostic and prognostic lncRNAs, we next used multivariate Cox regression analysis and built a model to assess the prognosis risk according to the following:

$$\text{Risk Score }(\text{RS})\;=\;\sum_{i=1}^{N}Exp_{i}*Coef_{i}$$

where *N* is the representative number of prognostic lncRNAs, *Exp_i_* is the expression value of the lncRNAs, and *Coef_i_* is a single factor Cox regression coefficient. *Risk Score (RS)* is the multinode weighted sum of risk scores.

### Statistical analysis

We adopted the median risk score as a cut-off value in the training dataset [22], using it to divide the osteosarcoma patients into low- and high-risk groups. We used Kaplan–Meier survival analysis to estimate recurrence time and compare recurrence curves between the two groups, and a two-sided log-rank test to assess the statistical significance. We then validated the prognostic ability of the lncRNA signature in the test dataset using the receiver operating characteristic (ROC) values and Kaplan–Meier survival analysis. We carried out data stratification analysis and multivariate Cox regression analysis in order to identify whether the lncRNA were an independent factor, and to assess the signature within the available data. We considered a value of *P*<0.05 as significant. All analyses were performed with the R statistical program (version 3.5.1), using the survival and pROC packages downloaded from Bioconductor (http://www.bioconductor.org/).

### Construction of the lncRNA-function network

The Kyoto Encyclopedia of Genes and Genomes pathway (KEGG) and gene ontology functional enrichment analyses (GO) were carried out to better illustrate the potential functional mechanism of recurrence-related lncRNA modules of osteosarcoma. The DAVID Bioinformatics Tool (https://david.ncifcrf.gov/), which consists of an integrated biological information base and analytical tools aimed at systematically extracting biological meaning from gene/protein lists was used. We first screened the three lncRNAs correlated with differentially expressed mRNAs (|Pearson correlation coefficient| > 0.4 and *P*-value < 0.05), and then screened the mRNAs involved in the functional analysis. These database inputs and screening parameters allowed for construction of the lncRNA-function network using Cytoscape, version.3.8.5 (https://cytoscape.org/).

## Results

### Patients’ characteristics

**Figure 1:**
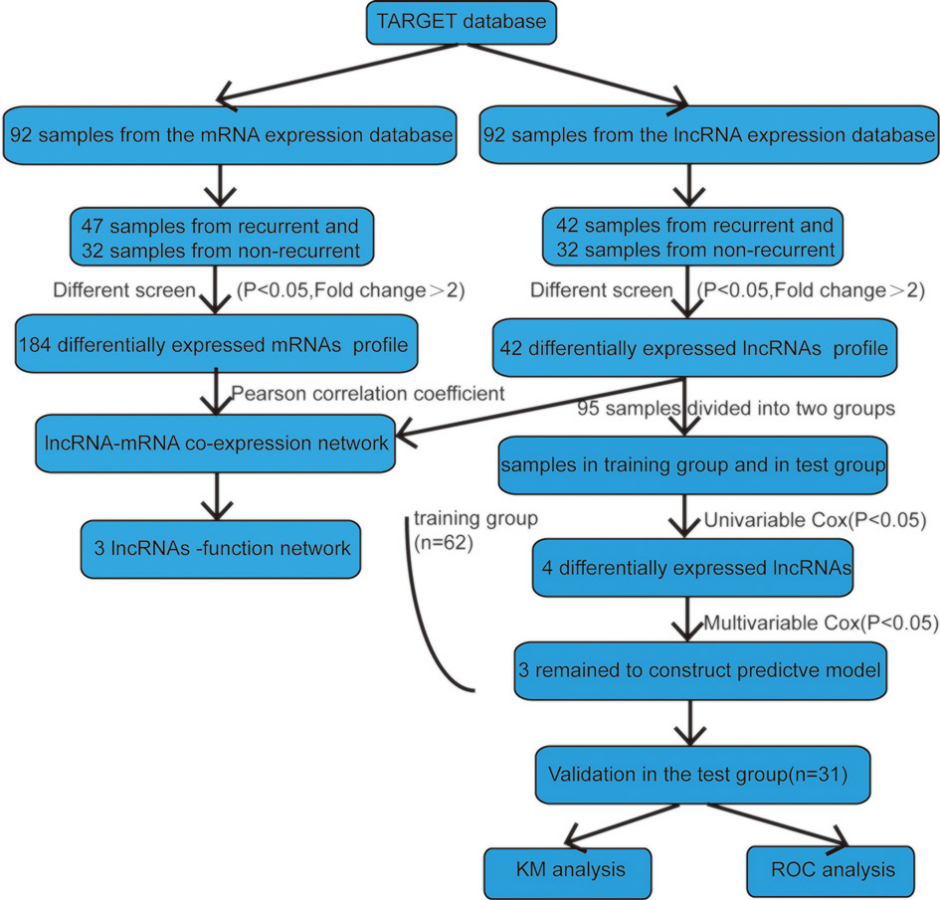
Flowchart of the study The order of analyses to develop the risk score model and validate the lncRNA signature to predict recurrence outcomes.

All expression data used in the present study were from patients clinically and pathologically diagnosed with osteosarcoma. The median age was 15 years (4–40 years) for the enrolled patients. Other clinical information about the patients is in Table 1. Subsequently, we randomly divided the 92 patients into two groups (training group, *n*=61; test group, *n*=31) to seek and validate the prognostic lncRNA found in the osteosarcoma patients. The selection scheme of the recurrent lncRNA signature can be found in Figure 1.

**Table 1:** Summary of patient demographics and characteristics

Characteristic	Training set	Testing set	Total
**Gender**			
Male	37	15	160
Female	24	16	27
**Age**			
Median	15	15	15
Range	4–40	6–21	4–40
**Vital status**			
Non-recurrence	28	16	44
Recurrence	33	15	48

### Identification of significantly differentially expressed mRNAs and lncRNAs

A total of 19495 mRNAs and 14589 lncRNAs were identified from the TARGET database. Using |Fold changes| ≥2 and *P*<0.05 as cutoffs, we identified 184 differentially expressed mRNAs (71 down-regulated and 113 up-regulated) and 42 differentially expressed lncRNAs (22 down-regulated and 20 up-regulated) (Supplementary Table S1).

### Construction of the lncRNA-mRNA co-expression network in osteosarcoma

**Figure 2:**
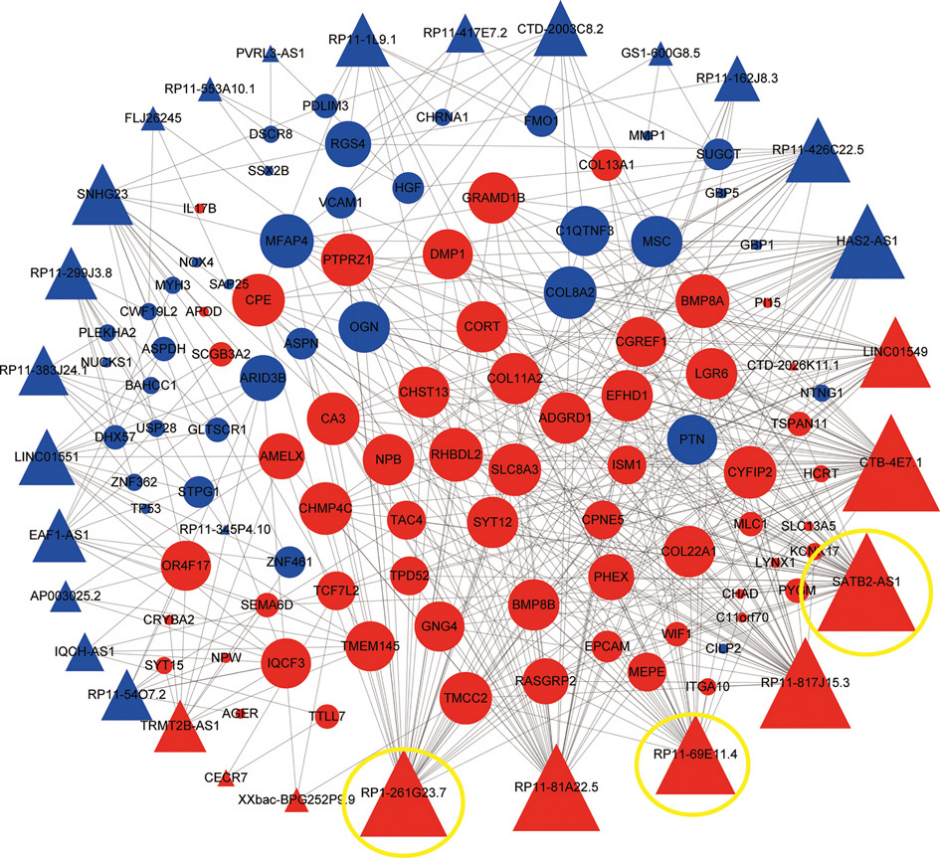
The lncRNA-mRNA co-expression network The lncRNA-mRNA co-expression network. Red balls represent up-regulated mRNAs and red cones represent up-regulated lncRNAs. Blue balls represent down-regulated mRNAs and blue cones represent down-regulated lncRNAs.

In order to better study the role of differentially expressed lncRNAs in osteosarcoma and to further elucidate the interaction between these differentially expressed mRNAs, we constructed an osteosarcoma lncRNA-mRNA related regulatory network. We used the 36 differentially expressed lncRNAs and 101 differentially expressed mRNAs to construct this network (Figure 2 and Supplementary Table S2), and screening criteria were the |Pearson correlation coefficient| > 0.4 and *P*-value <0.05. Using these criteria, we found that six up-regulated lncRNAs (RP1-261G23.7, RP11-81A22.5, RP11-69E11.4, RP11-817J15.3, SATB2-AS1 and CTB-4E7.1) functioned in regulating many mRNAs, suggesting an important regulatory role for these lncRNAs in osteosarcoma.

### Construction of the recurrent lncRNA signature with the training dataset

The training group (*n*=61), which included complete clinical information, was used to explore the association of recurrence with 42 differentially expressed lncRNAs. We initially performed a univariate Cox proportional hazards regression analysis of the expression profiling data of differentially expressed lncRNAs, with recurrence time and recurrence status as the dependent variables. We identified four lncRNAs that were significantly correlated with recurrence of patients (*P*-value <0.05, Supplementary Table S3). In order to screen the most powerful diagnostic and prognostic lncRNAs, we next used a multivariate Cox regression analysis (Supplementary Table S4) and built a 3-lncRNA (RP1-261G23.7, RP11-69E11.4, and SATB2-AS1) model to assess the recurrence risk. This set of 3-lncRNAs also functions in regulating the lncRNA-mRNA co-expression network. The risk score (Supplementary Table S5) of the combination of RP1-261G23.7, RP11-69E11.4, and SATB2-AS1 was determined as follows:

$$\text{RS}=(0.01\times ev_{RP1-261G23.7})+(0.01\times ev_{RP11-69E11.4})+(0.02\times ev_{SATB2-AS1})$$

where *RS* is the risk score and *ev* is the expression value.

### Determining the survival power of the lncRNA signature in the training and test datasets

**Figure 3:**
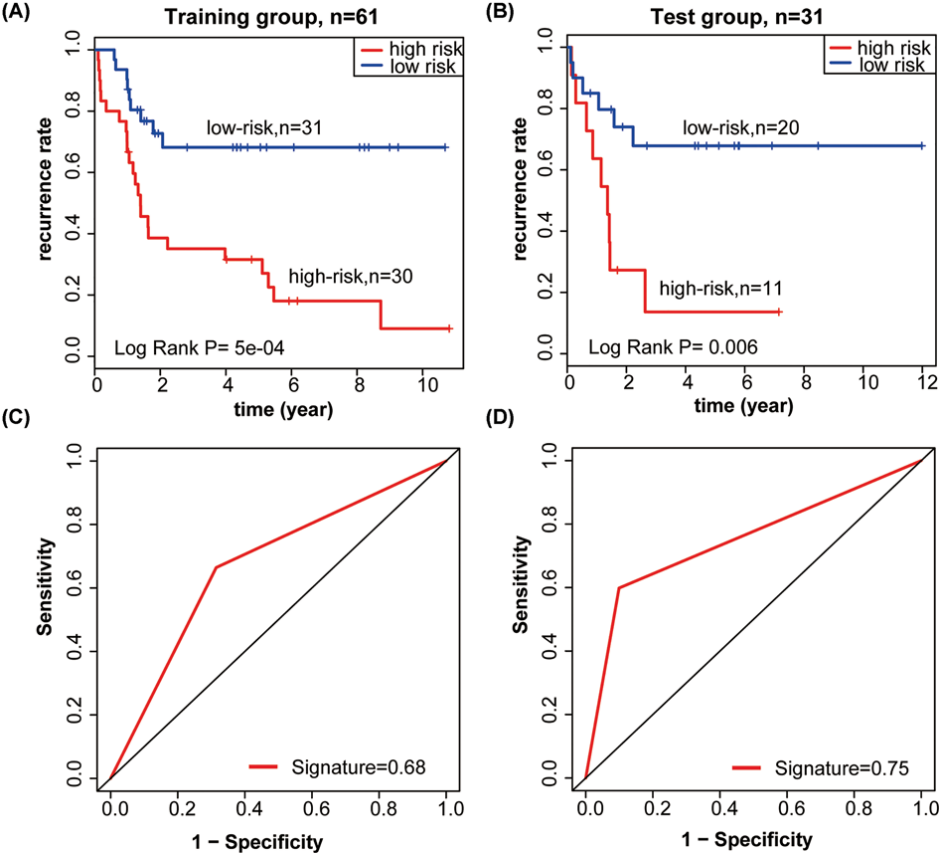
lncRNA signature predicts recurrence of patients with osteosarcoma (**A,B**) Kaplan–Meier survival curves classified osteosarcoma patients into high-risk and low-risk groups using the lncRNAs signature in the training and test datasets. *P*-values were calculated by log-rank test. (**C,D**) Results of ROC analysis.

The selected lncRNA signature returned a risk score for each patient. We used the median risk score to divide the training group patients into either a low-risk group (*n*=31) or a high-risk group (*n*=30). Using the Kaplan–Meier survival analysis, we found that the low-risk group patients had a significantly lower recurrence rate than those in the high-risk group (median recurrence time: >128.22 vs. 16.80 months, log-rank test *P*<0.001; Figure 3A). The 5-year recurrence rate of the patients in the high-risk group was less than 20%, while that of the patients in the low-risk group was greater than 60%.

The recurrent risk score model used to calculate the lncRNA signature-based risk scores of the 31 patients in the test dataset was also used to validate the prediction power of the lncRNA signature. Patients were divided into low-risk and high-risk groups using the median risk score as a demarcation point, in the same fashion as the training dataset. Kaplan–Meier curves were used to show the low-risk and high-risk groups in the test dataset (Figure 3B), and the results were similar to those from the training dataset. In the test dataset, the low-risk group had a significantly higher recurrence rate than the high-risk group (median recurrence time: >143.80 vs. 16.32, log-rank test *P*=0.006). The recurrence rate of patients in the high-risk group was less than 20% at 5 years, in contrast with more than 60% in the low-risk group.

### The survival prediction power of the lncRNA signature in the training and test groups

We performed a separate ROC analysis to test the prediction power of the lncRNA signature, which considered the larger area under the ROC curve as a better model for predicting recurrence of the osteosarcoma. In the training dataset, the predictive ability of the 3-lncRNA signature was high (AUC_Signature_ = 0.68, Figure 3C), which further demonstrated that this signature was a novel and highly accurate biomarker of recurrence. A similar result was seen in the test group (AUC_Signature_ = 0.75, Figure 3D).

### The selected 3-lncRNA signature is an independent prognostic factor

A multivariate Cox regression analysis using the signature-based risk score and other clinical characteristics (e.g. age and gender) demonstrated that the prognostic power of the lncRNA signature risk score for the prediction of recurrence was independent of clinical characteristics for the entire dataset (High-risk group vs. Low-risk group, HR = 2.06, 95% CI: 1.53–2.77, *P*<0.001, *n*=92, Table 2).

**Table 2:** Univariable and multivariable Cox regression analysis of the association between the 3-lncRNA signature and the recurrence of osteosarcoma patients (*n*=91)

Variables		HR	95% CI of HR		*P*
			Lower	Upper	
Univariable analysis					
Gender	Male vs. female	1.24	0.69	2.21	0.47
Age	>15 vs. ≤15	0.96	0.90	1.02	0.17
LncRNA signature	High risk vs. low risk	2.10	1.57	2.81	0.00
Multivariable analysis					
Gender	Male vs. female	1.17	0.63	2.16	0.62
Age	>15 vs. ≤15	0.96	0.89	1.02	0.18
LncRNA signature	High risk vs. low risk	2.06	1.53	2.77	0.00

### Functional prediction of differentially expressed mRNAs related to 3 lncRNAs

**Figure 4:**
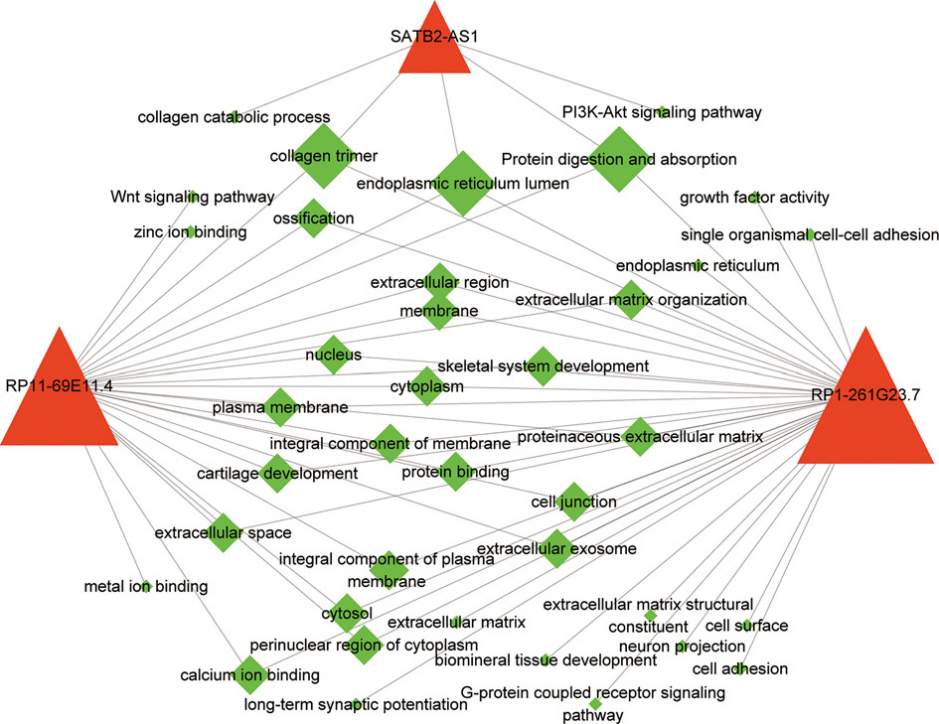
The lncRNA-function network The lncRNA-function network. Red cones represent lncRNAs and green diamonds represent functions.

KEGG and GO analyses were used to investigate the potential involvement of the 3 lncRNAs in biological processes associated with osteosarcoma development. We performed functional analysis with co-expressed mRNAs of 3 lncRNAs in each lncRNA module (|Pearson correlation coefficient| > 0.4 and *P*-value <0.05, Supplementary Table S6). The 3 lncRNAs-functional network (Figure 4 and Supplementary Table S6) was created using Cytoscape. The results indicated that the 3 lncRNA signature was related to a collagen trimer, endoplasmic reticulum lumen, and protein digestion and absorption. We also found that only RP1-261G23.7 was involved in the Wnt signal pathway, which is in a variety of biological processes, including cell cycle regulation and cancer. RP11-69E11.4 was involved in the G-protein coupled receptor signaling pathway, which is linked to the activation of adenylyl cyclase activity and subsequent cAMP/PKA/CREB signaling [23] as potential drivers of tumorigenesis. Finally, SATB2-AS1 is involved in the PI3K-Akt signaling pathway, which is essential in cell proliferation and the biological characteristics of malignant cells [24].

## Discussion

Individuals with recurrent osteosarcoma have less than a 20% chance of long-term survival, despite aggressive therapies [25]. Identification of a clinically relevant signature for prognosis is of critical need for patients. Recent studies suggested that lncRNAs could be good candidates for tumor signatures as they possess high sensitivity and high specificity [26–30]. Increasing data have demonstrated that lncRNAs are associated with osteosarcoma prognosis. For example, up-regulated FOXC2-AS1 was correlated with longer survival time [31], while HULC was associated with distant metastasis and clinical stage, and with shorter overall survival time [32,33]. Moreover, BANCR was associated with large tumor size, distant metastasis, and advanced clinical stage, and was an independent predictor of poor survival rate [34–36]. However, lncRNA signatures and their relation to recurrence of osteosarcoma has rarely been investigated.

In our study, different statistical methods were used to identify a 3-lncRNA signature associated with the recurrence of osteosarcoma in patients. We found that the lncRNA signature was an independent predictor in patient recurrence. A multivariate Cox regression analysis was used to assess the independence of the selected signature in predicting recurrence of osteosarcoma. With age and gender as covariables, the risk scores of patients based on the signature maintained an independent association with recurrence. Taken together, these results suggested that the prognostic power of the lncRNA signature for predicting recurrence of osteosarcoma in patients was independent of other clinical characteristics.

Moreover, the expression levels of RP1-261G23.7, RP11-69E11.4, and SATB2-AS1 were increased in recurrent osteosarcoma samples vs. non-recurrent samples. These lncRNAs also had important regulatory roles in osteosarcoma based on the lncRNA-mRNA co-expression network findings. Finally, we analyzed the functions of these lncRNAs, and the results showed that RP1-261G23.7 was involved in growth factor activity, single organismal cell–cell adhesion, the G-protein coupled receptor signaling pathway, and cell adhesion.

Vascular endothelial growth factor A (VEGF-A) is known to function directly in blood vessel formation and maintenance, and is a promising therapeutic target for activation in cancer. RP1-261G23.7 is antisense VEGF-A lncRNA, and a recent report showed that RP1-261G23.7 functions as a VEGF-A promoter enhancer-like element, possibly by acting as a local scaffold for proteins [37]. RP11-69E11.4 was involved in the Wnt signaling pathway, which is sensitive to alterations at various nodes of the pathway, and these alterations were identified in multiple tumor types after hyperactivation. These alterations converge into increased tumorigenicity, sustained proliferation and enhanced metastatic potential [24]. In addition, SATB2-AS1 is specifically involved in the PI3K-Akt signaling pathway, which can affect the epithelial–mesenchymal transition in numerous ways to influence tumor aggressiveness [38]. SATB2-AS1 was reported as overexpressed in osteosarcoma, and was associated with increased cell proliferation and growth [39]. These results indicated that recurrent lncRNA signatures executed a variety of biological functions in osteosarcoma tumorigenesis and development. There has been relatively little research on the three signature lncRNAs, and our results provide a foundation for further investigation into the function of the three lncRNAs.

There are a few limitations of this research regarding osteosarcoma. Most importantly, the specific prediction mechanism of the 3 lncRNAs in osteosarcoma requires further study. Moreover, the lncRNA signature has not yet been tested prospectively in multiple clinical trials. Despite these drawbacks, the significant and consistent correlation of our lncRNA signature with recurrence in two independent datasets indicated that it was a potentially powerful prognostic signature for osteosarcoma.

In conclusion, our data clearly indicate that the lncRNA signature can predict the recurrence of osteosarcoma in patients. The signature provides accurate prediction, which is clinically very significant.

## Supplementary Material

Supplementary Tables S1-S6

## Abbreviations

GO: gene ontology
KEGG: Kyoto Encyclopedia of Genes and Genomes
lncRNA: long noncoding RNA
ROC: receiver operating characteristic
TARGET: Therapeutically Applicable Research To Generate Effective Treatments

## References

[1] Tian W., Li Y., Zhang J., Li J. and Gao J. (2018) Combined analysis of DNA methylation and gene expression profiles of osteosarcoma identified several prognosis signatures. Gene 650, 7–14 10.1016/j.gene.2018.01.093 29407229

[2] Yu D., Kahen E., Cubitt C.L., McGuire J., Kreahling J., Lee J. et al. (2015) Identification of synergistic, clinically achievable, combination therapies for osteosarcoma. Sci. Rep. 5, 16991 10.1038/srep16991 26601688 PMC4658502

[3] Ba Z., Gu L., Hao S., Wang X., Cheng Z. and Nie G. (2018) Downregulation of lncRNA CASC2 facilitates osteosarcoma growth and invasion through miR-181a. Cell Prolif. 51, 1 10.1111/cpr.12409 29194827 PMC6528952

[4] Ye H., Lin J., Yao X., Li Y., Lin X. and Lu H. (2018) Overexpression of long non-coding RNA NNT-AS1 correlates with tumor progression and poor prognosis in osteosarcoma. Cell. Physiol. Biochem. 45, 1904–1914 10.1159/000487966 29518771

[5] Yan L., Wu X., Yin X., Du F., Liu Y. and Ding X. (2018) LncRNA CCAT2 promoted osteosarcoma cell proliferation and invasion. J. Cell. Mol. Med. 22, 2592–2599 10.1111/jcmm.13518 29502343 PMC5908115

[6] Li Z., Dou P., Liu T. and He S. (2017) Application of long noncoding RNAs in osteosarcoma: biomarkers and therapeutic targets. Cell. Physiol. Biochem. 42, 1407–1419 10.1159/000479205 28715796

[7] Dong Y., Liang G., Yuan B., Yang C., Gao R. and Zhou X. (2015) MALAT1 promotes the proliferation and metastasis of osteosarcoma cells by activating the PI3K/Akt pathway. Tumour Biol. 36, 1477–1486 10.1007/s13277-014-2631-4 25431257

[8] Zhou S., Yu L., Xiong M. and Dai G. (2018) LncRNA SNHG12 promotes tumorigenesis and metastasis in osteosarcoma by upregulating Notch2 by sponging miR-195-5p. Biochem. Biophys. Res. Commun. 495, 1822–1832 10.1016/j.bbrc.2017.12.047 29229388

[9] Zhang Q., Geng P.L., Yin P., Wang X.L., Jia J.P. and Yao J. (2013) Down-regulation of long non-coding RNA TUG1 inhibits osteosarcoma cell proliferation and promotes apoptosis. Asian Pac. J. Cancer Prev. 14, 2311–2315 10.7314/APJCP.2013.14.4.2311 23725133

[10] Tian Z.Z., Guo X.J., Zhao Y.M. and Fang Y. (2015) Decreased expression of long non-coding RNA MEG3 acts as a potential predictor biomarker in progression and poor prognosis of osteosarcoma. Int. J. Clin. Exp. Pathol. 8, 15138–15142 26823857 PMC4713643

[11] Zhou M., Wang X., Shi H., Cheng L., Wang Z., Zhao H. et al. (2016) Characterization of long non-coding RNA-associated ceRNA network to reveal potential prognostic lncRNA biomarkers in human ovarian cancer. Oncotarget 7, 12598–12611 26863568 10.18632/oncotarget.7181 PMC4914307

[12] Ma B., Li M., Zhang L., Huang M., Lei J.B., Fu G.H. et al. (2016) Upregulation of long non-coding RNA TUG1 correlates with poor prognosis and disease status in osteosarcoma. Tumour Biol. 37, 4445–4455 10.1007/s13277-015-4301-6 26499949

[13] Xu R., Feng F., Yu X., Liu Z. and Lao L. (2018) LncRNA SNHG4 promotes tumour growth by sponging miR-224-3p and predicts poor survival and recurrence in human osteosarcoma. Cell Prolif. 51, e12515 10.1111/cpr.12515 30152090 PMC6528889

[14] Cai L., Lv J., Zhang Y., Li J., Wang Y. and Yang H. (2017) The lncRNA HNF1A-AS1 is a negative prognostic factor and promotes tumorigenesis in osteosarcoma. J. Cell. Mol. Med. 21, 2654–2662 10.1111/jcmm.12944 28866868 PMC5661255

[15] Robinson M.D., McCarthy D.J. and Smyth G.K. (2010) edgeR: a Bioconductor package for differential expression analysis of digital gene expression data. Bioinformatics 26, 139–140 10.1093/bioinformatics/btp616 19910308 PMC2796818

[16] Xiong H.G., Li H., Xiao Y., Yang Q.C., Yang L.L., Chen L. et al. (2019) Long noncoding RNA MYOSLID promotes invasion and metastasis by modulating the partial epithelial-mesenchymal transition program in head and neck squamous cell carcinoma. J. Exp. Clin. Cancer Res. 38, 278 10.1186/s13046-019-1254-4 31238980 PMC6593600

[17] Larsen T.V., Hussmann D. and Nielsen A.L. (2019) PD-L1 and PD-L2 expression correlated genes in non-small-cell lung cancer. Cancer Commun. (Lond.) 39, 30 10.1186/s40880-019-0376-6 31159869 PMC6545701

[18] Wang B., Ran Z., Liu M. and Ou Y. (2019) Prognostic significance of potential immune checkpoint member HHLA2 in human tumors: a comprehensive analysis. Front. Immunol. 10, 1573 10.3389/fimmu.2019.01573 31379814 PMC6644528

[19] Kawaguchi T., Azuma K., Sano M., Kim S., Kawahara Y., Sano Y. et al. (2017) The Japanese version of the National Cancer Institute’s patient-reported outcomes version of the common terminology criteria for adverse events (PRO-CTCAE): psychometric validation and discordance between clinician and patient assessments of adverse events. J. Patient Rep. Outcomes 2, 2 10.1186/s41687-017-0022-5 29757309 PMC5934922

[20] Bie L.Y., Li D., Mu Y., Wang S., Chen B.B., Lyu H.F. et al. (2019) Analysis of cyclin E co-expression genes reveals nuclear transcription factor Y subunit alpha is an oncogene in gastric cancer. Chronic Dis. Transl. Med. 5, 44–52 10.1016/j.cdtm.2018.07.003 30993263 PMC6449734

[21] Jeong H.H., Kim S., Wee K. and Sohn K.A. (2015) Investigating the utility of clinical outcome-guided mutual information network in network-based Cox regression. BMC Syst. Biol. 9, S8 10.1186/1752-0509-9-S1-S8 25708115 PMC4331683

[22] Zhou M., Guo M., He D., Wang X., Cui Y., Yang H. et al. (2015) A potential signature of eight long non-coding RNAs predicts survival in patients with non-small cell lung cancer. J. Transl. Med. 13, 231 10.1186/s12967-015-0556-3 26183581 PMC4504221

[23] Alakus H., Babicky M.L., Ghosh P., Yost S., Jepsen K., Dai Y. et al. (2014) Genome-wide mutational landscape of mucinous carcinomatosis peritonei of appendiceal origin. Genome Med. 6, 43 10.1186/gm559 24944587 PMC4062050

[24] Tai D., Wells K., Arcaroli J., Vanderbilt C., Aisner D.L., Messersmith W.A. et al. (2015) Targeting the WNT signaling pathway in cancer therapeutics. Oncologist 20, 1189–1198 10.1634/theoncologist.2015-0057 26306903 PMC4591954

[25] Ferrari S., Smeland S., Mercuri M., Bertoni F., Longhi A., Ruggieri P. et al. (2005) Neoadjuvant chemotherapy with high-dose Ifosfamide, high-dose methotrexate, cisplatin, and doxorubicin for patients with localized osteosarcoma of the extremity: a joint study by the Italian and Scandinavian Sarcoma Groups. J. Clin. Oncol. 23, 8845–8852 10.1200/JCO.2004.00.5785 16246977

[26] Shi T., Gao G. and Cao Y. (2016) Long noncoding RNAs as novel biomarkers have a promising future in cancer diagnostics. Dis. Markers 2016, 9085195 10.1155/2016/9085195 27143813 PMC4842029

[27] Yarmishyn A.A. and Kurochkin I.V. (2015) Long noncoding RNAs: a potential novel class of cancer biomarkers. Front. Genet. 6, 145 10.3389/fgene.2015.00145 25954300 PMC4407501

[28] Hu H.B., Jie H.Y. and Zheng X.X. (2016) Three circulating LncRNA predict early progress of esophageal squamous cell carcinoma. Cell. Physiol. Biochem. 40, 117–125 10.1159/000452529 27855375

[29] Qiu Z.L., Shen C.T., Sun Z.K., Wei W.J., Zhang X.Y., Song H.J. et al. (2016) Circulating long non-coding RNAs act as biomarkers for predicting 131I uptake and mortality in papillary thyroid cancer patients with lung metastases. Cell. Physiol. Biochem. 40, 1377–1390 10.1159/000453190 27997908

[30] Tang Q., Ni Z., Cheng Z., Xu J., Yu H. and Yin P. (2015) Three circulating long non-coding RNAs act as biomarkers for predicting NSCLC. Cell. Physiol. Biochem. 37, 1002–1009 10.1159/000430226 26393913

[31] Zhu K.P., Zhang C.L., Shen G.Q. and Zhu Z.S. (2015) Long noncoding RNA expression profiles of the doxorubicin-resistant human osteosarcoma cell line MG63/DXR and its parental cell line MG63 as ascertained by microarray analysis. Int. J. Clin. Exp. Pathol. 8, 8754–8773 26464619 PMC4583851

[32] Sun X.H., Yang L.B., Geng X.L., Wang R. and Zhang Z.C. (2015) Increased expression of lncRNA HULC indicates a poor prognosis and promotes cell metastasis in osteosarcoma. Int. J. Clin. Exp. Pathol. 8, 2994–3000 26045809 PMC4440118

[33] Uzan V.R., Lengert A., Boldrini E., Penna V., Scapulatempo-Neto C., Scrideli C.A. et al. (2016) High expression of HULC is associated with poor prognosis in osteosarcoma patients. PLoS ONE 11, e0156774 10.1371/journal.pone.0156774 27253450 PMC4890737

[34] Peng Z.Q., Lu R.B., Xiao D.M. and Xiao Z.M. (2016) Increased expression of the lncRNA BANCR and its prognostic significance in human osteosarcoma. Genet. Mol. Res. 4, 34 10.4238/gmr.15017480 27051014

[35] Flockhart R.J., Webster D.E., Qu K., Mascarenhas N., Kovalski J., Kretz M. et al. (2012) BRAFV600E remodels the melanocyte transcriptome and induces BANCR to regulate melanoma cell migration. Genome Res. 22, 1006–1014 10.1101/gr.140061.112 22581800 PMC3371703

[36] Fang D., Yang H., Lin J., Teng Y., Jiang Y., Chen J. et al. (2015) 17beta-estradiol regulates cell proliferation, colony formation, migration, invasion and promotes apoptosis by upregulating miR-9 and thus degrades MALAT-1 in osteosarcoma cell MG-63 in an estrogen receptor-independent manner. Biochem. Biophys. Res. Commun. 457, 500–506 10.1016/j.bbrc.2014.12.114 25592968

[37] Nieminen T., Scott T.A., Lin F.M., Chen Z., Yla-Herttuala S. and Morris K.V. (2018) Long non-coding RNA modulation of VEGF-A during hypoxia. Noncoding RNA 15, gmr7480 10.4238/gmr.15017480 30463374 PMC6315885

[38] Lunardi A., Webster K.A., Papa A., Padmani B., Clohessy J.G., Bronson R.T. et al. (2014) Role of aberrant PI3K pathway activation in gallbladder tumorigenesis. Oncotarget 5, 894–900 10.18632/oncotarget.1808 24658595 PMC4011591

[39] Liu S.H., Zhu J.W., Xu H.H., Zhang G.Q., Wang Y., Liu Y.M. et al. (2017) A novel antisense long non-coding RNA SATB2-AS1 overexpresses in osteosarcoma and increases cell proliferation and growth. Mol. Cell. Biochem. 430, 47–56 10.1007/s11010-017-2953-9 28190168

